# Rare Congenital Aberrant Left Superior Pulmonary Vein Discovered with Central Line Placement in a Patient with Critical Cardiorespiratory Collapse

**DOI:** 10.1155/2017/8728904

**Published:** 2017-09-13

**Authors:** Spencer Knox, Mario Madruga, S. J. Carlan

**Affiliations:** ^1^Orlando Regional Healthcare, Department of Medicine, Orlando, FL, USA; ^2^Orlando Regional Healthcare, Division of Academic Affairs and Research, Orlando, FL, USA

## Abstract

**Background:**

Partial anomalous pulmonary venous connection is a rare congenital vascular disorder that may be asymptomatic. Left-sided connections with the innominate vein are discovered infrequently and those without an atrial septal defect are extremely rare.

**Case:**

A 66-year-old male was found to have an anomalous left pulmonary vein when a central venous catheter was inserted for management of hypoxemia. In addition to the connection with the left innominate vein an echocardiogram revealed no atrial septal defect. Computed tomography arteriography was used to define the anomaly.

**Conclusion:**

Left superior vein partial anomalous pulmonary venous connection with the left innominate vein was discovered incidentally on insertion of central venous catheter. The otherwise innocuous anomaly can become a significant variable when treating critical cardiopulmonary collapse.

## 1. Introduction

An aberrant left superior pulmonary vein is a rare congenital defect that is typically asymptomatic and has little clinical significance. It is usually discovered incidentally with imaging for other reasons. The condition belongs to a group of cardiac anomalies known as partial anomalous pulmonary venous connection (PAPVC) in which from one to three out of four pulmonary veins drain into the right atrium or its tributaries. PAPVC has a documented incidence of 0.4–0.7% in autopsy specimens in the United States [1] with only 10% of anomalous pulmonary veins originating from the left side [2]. An intact atrial septum is rare [3]. The most common interatrial septal defect type associated with PAPVC is the sinus venosus [4]. Only 3% of patients are reported to have PAPVC from the left lung to the left innominate vein [5]. We report a case of PAPVC characterized by the left superior pulmonary vein entering directly into the left innominate vein in a patient with an intact atrial septum that was discovered during treatment for cardiopulmonary failure.

## 2. Case

**Figure 1:**
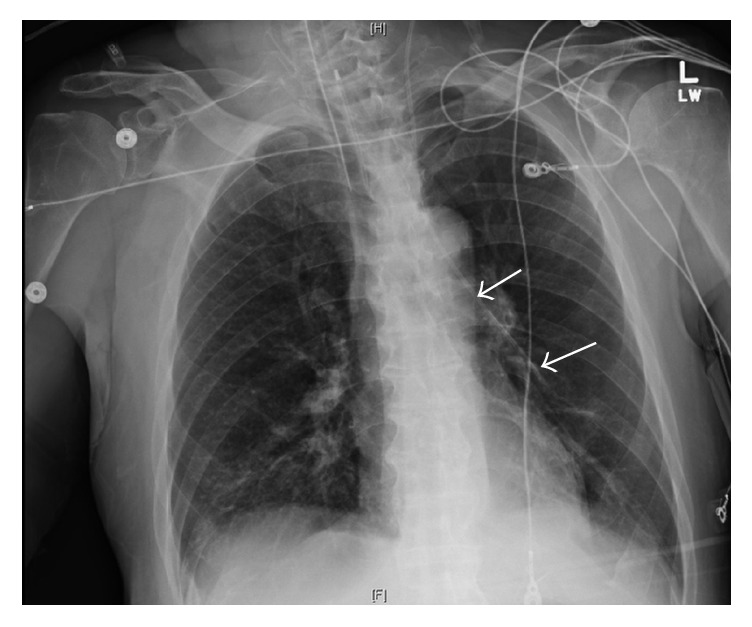
Inspiratory chest X-ray showing left internal jugular central catheter in aberrant left superior pulmonary vein (arrows).

**Figure 2:**
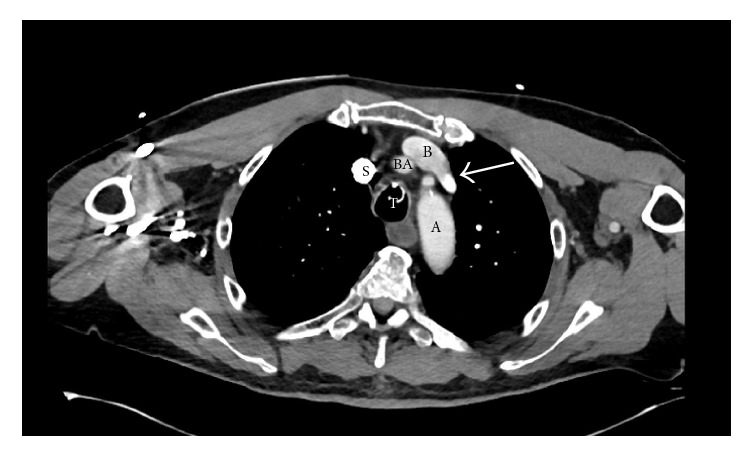
Cross section CTA of chest. S = superior vena cave, B = innominate vein, BA = innominate artery, T = trachea, and A = aorta. Arrow points to the insertion of aberrant left superior pulmonary vein into innominate vein.

A 66-year-old male with a history of hypertension, dyslipidemia, schizophrenia, dementia, and chronic obstructive pulmonary disease (COPD) presented to the emergency department ED for altered mental status, hypotension, and hypoxia. He was sent from inpatient hospice after receiving lorazepam, ziprasidone, and phenobarbital for agitation earlier in the day. The patient was evaluated for suspected acute hypoxemic respiratory failure with oxygen saturation in the 40%, marginally improving to 60% with nonrebreather mask. He was admitted for acute hypoxemic respiratory failure and subsequently underwent emergent intubation for persistent hypoxemia. Purulent material was also noted in the airway during intubation. Computed tomography (CT) of the head was negative. Computed tomography angiography (CTA) of the thorax revealed evidence of lower lobe bronchial obstruction likely due to secretions and was negative for pulmonary embolus. Subsequent bronchoscopy revealed patent airways to the subsegmental level with thin clear frothy secretions. Due to persistent hypotension in the setting of sepsis, a central venous (CV) catheter was placed via the left internal jugular approach with excellent nonpulsatile blood return. A subsequent follow-up chest X-ray performed immediately after the procedure revealed an abnormal anatomical position of the central catheter. Instead of passing into the innominate vein, the left internal jugular central catheter appeared to be radiologically located in the left hemithorax (Figure 1). On review of the previous CTA it was apparent that the patient had an aberrant left superior pulmonary vein draining into the left innominate vein (Figure 2). He had an echocardiogram showing no ASD but right atrial enlargement, abnormal left ventricular diastolic function, trace tricuspid insufficiency, and a mildly abnormal left ventricular ejection fraction at 45–49%. Our patient was started on a norepinephrine drip with expected effect. He improved with appropriate intravenous antibiotics, antipsychotic Olanzapine, including centrally acting agents donepezil and amantadine. He was discharged to a skilled nursing facility in stable condition. The vascular anomaly was felt to be clinically significant in the context of the compromised cardiopulmonary status.

## 3. Discussion

This case is unique for three reasons. First, because of the anatomic findings: our patient presented with an exceptionally infrequent variant of PAPVC, a left-sided aberrant superior pulmonary vein draining into the left innominate vein. Also, our patient's echocardiogram revealed a large right atrium without an atrial septal defect. This combination of structural findings is notable with less than a dozen previous reports for this anatomic arrangement [5–9].

Second is the circumstance surrounding the discovery of the anomaly. The patient was in the process of a workup for hypoxemia when a check chest X-ray demonstrated an abnormal central catheter tip placement. Review of the chest CTA from the previous day finally identified the anomaly, but the disorder probably would have remained unidentified without the catheter misplacement on the check chest X-ray. These disorders can be difficult to diagnose when they are clinically silent [10]. Transesophageal echocardiography or invasive cardiac catheterization can be helpful in certain cases.

Third, and most important, is the critical clinical condition of the patient and the potential effect the vascular anomaly had on his hemodynamic response to the respiratory collapse. It was unlikely that the dilated right atrium was an acute change considering that his left superior pulmonary vein had been delivering excess volume return to the right atrium via the innominate vein, his entire life. But when added to the acute collapse of his respiratory function it is not improbable that his compromised ejection fraction and abnormal diastolic function were elements of his clinical presentation and recovery that may have been aggravated by the combination of chronic right-sided overflow and acute hypoxemia. The underlying anomaly likely affected his hemodynamics adversely during his acute respiratory failure and thus could have compromised the treatment algorithm. Consequently, this vascular anomaly was benign until the system failed and then became a variable that added complexity and morbidity to the patient's presentation, treatment, and response. There are cases of left-sided PAPVC that are symptomatic and require surgical correction even before a catastrophic cardiopulmonary failure [11]. However, this case illustrates that a benign vascular anomaly can complicate the care of a critically ill patient and must be accounted for.
